# GLP-1 receptor agonists as promising anti-inflammatory agents in heart failure with preserved ejection fraction

**DOI:** 10.1007/s10741-024-10450-6

**Published:** 2024-10-19

**Authors:** Giovanni Battista Bonfioli, Luca Rodella, Marco Metra, Enrico Vizzardi

**Affiliations:** Cardiology, ASST Spedali Civili Di Brescia; Department of Medical and Surgical Specialties, Radiological Sciences, and Public Health, University of Brescia, Brescia, Italy

**Keywords:** Heart Failure with Preserved Ejection Fraction, GLP-1, Inflammation

## Abstract

Heart Failure with Preserved Ejection Fraction (HFpEF) represents a significant challenge in modern cardiovascular medicine, characterized by diastolic dysfunction and a chronic pro-inflammatory milieu. The high prevalence of comorbidities such as diabetes, visceral obesity, and aging, which contribute to systemic inflammation, plays a pivotal role in the pathogenesis and progression of HFpEF. Glucagon-Like Peptide-1 Receptor Agonists (GLP-1 RAs), a class of glucose-lowering drugs, have demonstrated a wide range of pleiotropic effects that extend beyond glycaemic control. These effects include the reduction of inflammation and oxidative stress, vasodilation, decreased arterial stiffness, and a reduction in myocardial fibrosis—key factors in the pathophysiology of HFpEF. Recent evidence from the STEP-HFpEF and STEP-HFpEF-DM trials provides the first robust data supporting the efficacy of GLP-1 RAs, specifically semaglutide, in improving the quality of life in obese patients with HFpEF. These trials also demonstrated a significant reduction in C-Reactive Protein (CRP) levels, reinforcing the hypothesis that suppressing the pro-inflammatory state may yield substantial clinical benefits in this patient population. These findings suggest that GLP-1 RAs could play a crucial role in the management of HFpEF, particularly in patients with obesity, by targeting the underlying inflammatory processes and contributing to better overall cardiovascular outcomes.

## Introduction

Inflammation is a well-known cardiovascular risk factor acting as a facilitator for atherosclerosis and atherosclerotic plaque rupture. Cardiovascular (CV) events are strongly related to interleukin-6 (IL-6) and C-reactive protein (CRP), independently from the classical CV risk factors [1–4]. The US Centers for Disease Control and Prevention and the American Heart Association have stated that people with CRP values in the upper tertile of the adult population (> 3.0 mg/L) have a risk of cardiovascular disease (CVD) that is double that of people whose CRP concentrations are less than 1.0 mg/L [5, 6].

Although inflammation is usually considered primarily associated to atherosclerotic CVD, it is also strongly bound to heart failure with preserved ejection fraction (HFpEF). HFpEF is characterized by the presence of different comorbidities such as type-2 diabetes mellitus (T2DM), visceral obesity and ageing, all contributing to systemic metabolic inflammation [7, 8]. Systemic and endothelial inflammation contribute to collagen deposition, interstitial fibrosis and therefore atrial and ventricular remodelling as well as vascular stiffening [9, 10]. This concept is supported by the reported elevation of CRP and other inflammatory biomarkers, as well as by evidence of inflammatory cells in endomyocardial biopsies of HFpEF patients [11–14].

In the last few years, different targeted therapies against pro-inflammatory cytokines in CVD were tested, but with no outstanding results. Colchicine and IL-1 inhibitor Canakinumab showed a reduction in major cardiovascular events in patients with previous myocardial infarction, but with no benefits on death by any cause [15–17]. TNF-α inhibitors such as Infliximab and Etanercept did not show benefits in chronic heart failure due to the worsening of the clinical status and some serious adverse effects, such as upper respiratory tract infections [18, 19]. Considering the high rate of side-effects, drugs with a more cardiovascular centred mechanism of action are being developed. Ziltivekimab, a new monoclonal antibody directed against IL-6, has been specifically designed for patients with atherosclerosis and high cardiovascular risk and is being evaluated both in patients with atherosclerosis and CKD and in patients with HFpEF [20–22].

## The anti-inflammatory properties of glucagon like peptide-1 receptor agonists

**Fig. 1:**
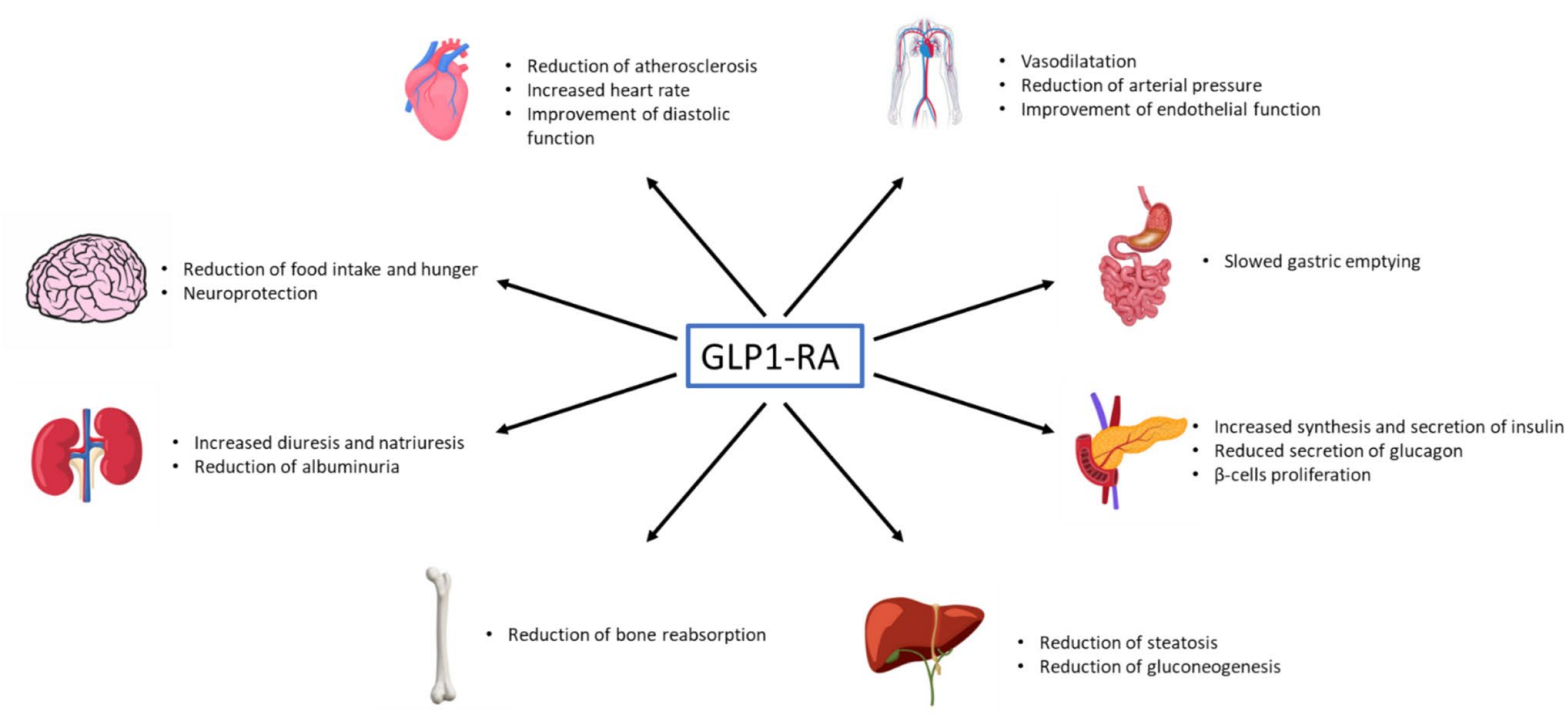
Visual representation of GLP1-RA pleotropic effects. GLP1-RA not only act on glycemic levels through the action on pancreatic β-cells, but also have many other effects that include weight loss both for a local action of slowed gastric emptying, but also for a central action of a reduction of hunger, and also cardiovascular, renal and hepatic protection

The glucagon like peptide-1 receptor agonists (GLP-1 RAs) are a glucose-lowering class of drugs that acts by mimicking the native GLP1 action, a peptide hormone synthesized by bowel endocrine L-cells and physiologically degraded by dipeptidyl-peptidase 4. They have an incretin action, reducing glucose plasmatic levels through the stimulation of insulin secretion by the pancreatic beta-cells and the concomitant inhibition of glucagon secretion by alpha-cells. GLP-1 RAs do not only act on insulin secretion but also have a wide spectrum of pleotropic action that is reported in Fig. 1. In particular, they have shown a reduction of major adverse cardiovascular events (MACE) in diabetic patients at high cardiovascular risk [23–30]. Therefore, they are nowadays recommended as first line therapy in patients with T2DM and atherosclerotic cardiovascular disease to reduce events, regardless of baseline or target HbA1c and concomitant glucose-lowering medication according to the 2023 ESC guidelines for the management of cardiovascular disease in patients with diabetes [31]. The reduction of cardiovascular events is not only related to anti-diabetic properties, but also to pleotropic effects leading to the reduction of the residual cardiovascular risk, that has been defined as the risk of progression of CV events (MACE or HF) that remains after the optimal glycaemic control in T2DM patients, and it seems to be related to meta-inflammation, defined as a low-grade chronic and sterile inflammatory status. Reduction of metabolic systemic inflammation could explain the reduction of CV events beyond glycaemic control with GLP-1 RA therapy.

The reduction of meta-inflammation may be also related to weight loss: GLP-1 RAs can slow the gastric emptying acting on the autonomic nervous system and reduce hunger through an action on the central nervous system (CNS). Both these effects contribute to weight loss during GLP-1 RA therapy and consequently to a decrease of the inflammatory burden thanks to the decline in adiponectin [6, 32]. Weight loss has been associated with a reduction in the risk for HFpEF or an attenuation of disease severity among patients with prevalent disease [33, 34]. Furthermore, obese patients have a higher quantity of epicardial adipose tissue (EAT). EAT can have some benefits such as thermogenic function, heart’s mechanical protection and secretion of cardioprotective adipokines (i.e. adiponectin). However, when it is in excess, these benefits are overcome by a pro-inflammatory activity, being associated with worse haemodynamic and metabolic profile in patients affected by HFpEF [35, 36]. Iacobellis et al. demonstrated that weekly administration of either GLP-1 RA semaglutide or dulaglutide produces a rapid, substantial and dose-dependent reduction in EAT thickness [37], that may bring to a reduction in the pro-inflammatory stimuli.

**Fig. 2:**
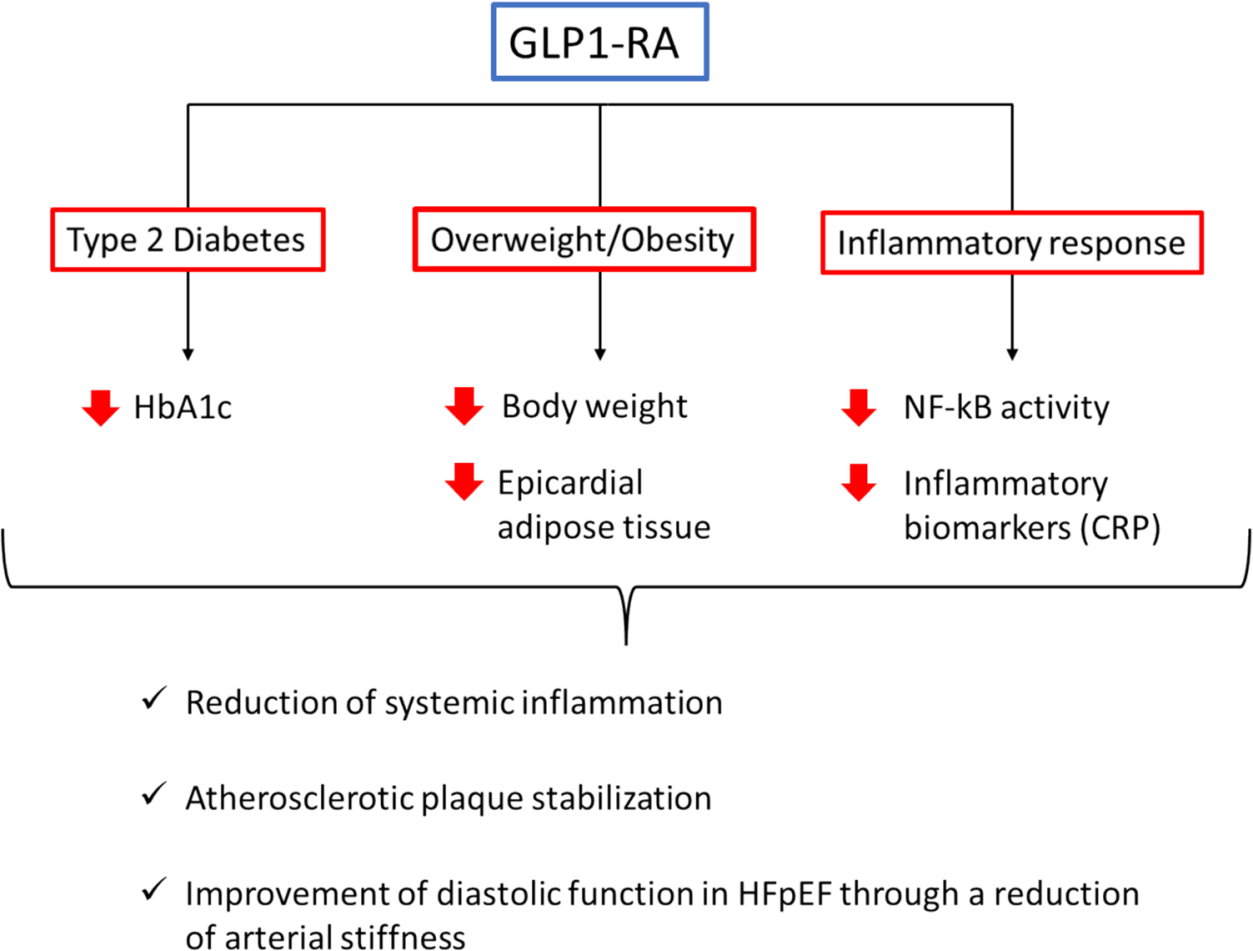
GLP-1 RAs may lead to a reduction of systemic inflammation through different mechanisms that include: (i) their direct effects on glycaemic levels; (ii) the reduction of body weight, epicardial adipose tissue and consequently of pro-inflammatory adipokines; (iii) the reduction of inflammatory response through different mechanisms as the reduction of the activity of NF-kB pattern and the reduction of different inflammatory biomarkers such as CRP. The reduction of systemic inflammatory response is associated with atherosclerotic plaque stabilization and improvement of diastolic function in HFpEF

The correlation between inflammation and GLP-1 level is complicated: it was shown that endogen GLP-1 is elevated in inflammatory settings, such as critically ill patients or after cardiac surgery. Many studies showed that GLP-1 RA in vitro can inhibit the inflammation and modulate immune response [38–42]. GLP-1 RAs may attenuate inflammatory response by down-regulating NF-kB signalling; studies in kidney tissue and in animal models showed a reduction of NF-kB activity after the administration of exendin-4 [43, 44]. Furthermore, GLP-1 RAs exhibit vasodilatory effects by promoting endothelial nitric oxide (NO) synthesis, enhancing endothelial function with a consequent reduction of vascular resistance and an improvement in cardiac afterload [45, 46]. A summary of GLP-1 RAs effects on inflammatory patterns is reported in Fig. 2.

## Clinical trials

GLP-1 RAs are an antidiabetic class of drugs, but their effect on the relative reduction in HbA1c compared to placebo was moderate (0.4–1.2%) in the outcome trials. As previously reported, their effect on MACE goes beyond glycaemic control: the reduction of atherosclerotic plaque formation, regardless HbA1c reduction, was confirmed by different studies on liraglutide and semaglutide. GLP-1 and GLP-1 RA (such as Exenatide and Liraglutide) were shown to have an anti-inflammatory action by reducing monocyte adhesion and macrophage accumulation, thus stabilizing atherosclerotic plaque, and slowing atherosclerosis in animal models [47–49]. A metanalysis by Bray et al. performed on 40 randomized clinical trials including T2DM patients showed that GLP1-RAs reduce oxidative stress and the levels of inflammatory markers including CRP [50]. As previously reported, the reduction of inflammation may be related to weight loss, that was significant in the different outcome trials, going from a 0.6-kg reduction at 12 weeks with Lixisenatide in ELIXA trial to 4.3 kg at 2 years with injectable semaglutide in SUSTAIN-6 trial [25, 26].

Regarding the role of GLP-1 RA in heart failure, some older trials did not show benefits in patients with HFrEF. In the FIGHT trial on patients hospitalized for HFrEF, Liraglutide did not show benefit on time to death, time to rehospitalization for heart failure, and time-averaged proportional change in N-terminal pro-B-type natriuretic peptide (NT-proBNP) levels from baseline to 180 days [51]. In the LIVE trial, Liraglutide did not show an improvement in left ventricular ejection fraction (LVEF) in a 24-week period in ambulatory patients affected by HF LVEF lower than 45%. However, a significant reduction in left atrial volume [mean difference − 8.7 mL (− 14.2, − 3.2), P = 0.002] and in E/e’ ratio [mean difference − 1.4 (− 2.7, − 0.1), P = 0.03] [52] was reported. Left atrial volume and E/e’ ratio are crucial parameters in HFpEF and their improvement with GLP1-RA, together with the reduction of inflammation markers, may explain the expected benefits of this class of drugs in patients affected by HFpEF.

The recent STEP-HFpEF trial showed that in patients with heart failure with preserved ejection fraction and obesity (BMI > 30), treatment with subcutaneous semaglutide (2.4 mg) led to larger reductions in symptoms and physical limitations, greater improvements in KCCQ and exercise function, and greater weight loss (− 10.7%) than placebo. Furthermore, there was a significant reduction of CRP level with semaglutide (− 43.5% vs − 7.3% with placebo, estimated treatment ratio, 0.61; 95% CI, 0.51 to 0.72; *P* < 0.001) [53]. The STEP-HFpEF-DM trial confirmed the benefits of once-weekly semaglutide also in obese patients affected by type 2 diabetes. Even in this last trial, CRP levels were significantly reduced compared to placebo (respectively − 42.0% vs − 12.8%, estimated treatment ratio 0.67; 95% C.I. 0.55 to 0.80, *P* < 0.001) [54]. These data were confirmed in a pooled analysis of the two trials with a reduction of CRP level with semaglutide of − 43% vs − 10% with placebo (OR 0.64, 95% C.I. 0.56 to 0.72, *P* < 0.001) [55].

## Future directions

The reduction of inflammation is of fundamental importance in HFpEF treatment, as CRP was associated both with cardiovascular mortality and all-cause mortality in a recent meta-analysis [56]. Thanks to their anti-inflammatory action through different mechanisms, GLP1-RA may provide, in a selected cohort of patients, the cardiovascular protection desired with targeted therapies, but without the wide side-effects of those drugs.

In the last years, many trials failed to find a unique treatment for all HFpEF phenotypes, apart from sodium-glucose cotransporter 2 inhibitors. As HFpEF is a complex and multifactorial disease and therefore a more targeted treatment based on individual comorbidities, it may change the natural history of the disease [57].

Despite the promising potential of GLP-1 RAs, it is essential to conduct further clinical studies to confirm their long-term efficacy and safety in patients with HFpEF. Although preliminary data are encouraging, especially regarding the reduction of inflammation and body weight, open questions remain regarding the effects of these drugs on a longer time scale and on different patient subgroups.

Future research should evaluate the impact of GLP-1 RAs on long-term clinical outcomes, such as cardiovascular mortality and hospitalizations for heart failure. It will also be important to determine whether these drugs can be used in combination with other therapies to optimize the treatment of HFpEF in obese patients, while minimizing side effects and drug interactions.

## Conclusion

GLP-1 RAs are emerging as a particularly promising class of drugs for the targeted treatment of obese patients with HFpEF. This subgroup of patients represents a significant clinical challenge due to the complexity of the disease, often burdened by several different comorbidities. GLP-1 RAs offer a potential benefit in these patients due to their pleiotropic effects, which go beyond simple glycemic control. Their anti-inflammatory action is essential, since low-grade inflammation is a key factor in the perpetuation of cardiovascular damage in patients with HFpEF, especially in the presence of obesity. Obesity-related chronic inflammation contributes not only to the alteration of cardiac metabolism, but also to the accumulation of epicardial adipose tissue, which can aggravate the hemodynamic profile and worsen cardiac function.

In summary, GLP-1 RAs represent a promising therapeutic option for obese patients with HFpEF, but their large-scale adoption will require further evidence of efficacy, safety, and long-term benefits through rigorous and well-designed clinical trials.

## Data Availability

No datasets were generated or analysed during the current study.
